# Predictors of short-term anxiety outcome in subthalamic stimulation for Parkinson’s disease

**DOI:** 10.1038/s41531-024-00701-6

**Published:** 2024-06-08

**Authors:** Anna Sauerbier, Johanna Herberg, Vasilija Stopic, Philipp A. Loehrer, Keyoumars Ashkan, Alexandra Rizos, Stefanie T. Jost, Jan Niklas Petry-Schmelzer, Alexandra Gronostay, Christian Schneider, Veerle Visser-Vandewalle, Julian Evans, Christopher Nimsky, Gereon R. Fink, Angelo Antonini, Pablo Martinez-Martin, Monty Silverdale, Daniel Weintraub, Anette Schrag, K. Ray Chaudhuri, Lars Timmermann, Haidar S. Dafsari, EUROPAR, the German Parkinson Society Non-motor Symptoms Study Group, and the International Parkinson and Movement Disorders Society Non-Motor Parkinson’s Disease Study Group, Charles Adler, Roongroj Bhidayasiri, Per Borghammer, Paolo Barone, David J. Brooks, Richard Brown, Marc Cantillon, Camille Carroll, Miguel Coelho, Cristian Falup-Pecurariu, Tove Henriksen, Michele Hu, Peter Jenner, Beomseok Jeon, Milica Kramberger, Padma Kumar, Mónica Kurtis, Valentina Leta, Simon Lewis, Irene Litvan, Kelly Lyons, Davide Martino, Mario Masellis, Hideki Mochizuki, James F. Morley, Melissa Nirenberg, Per Odin, Javier Pagonabarraga, Jalesh Panicker, Nicola Pavese, Eero Pekkonen, Ron Postuma, Mayela Rodriguez Violante, Raymond Rosales, Anthony Schapira, Tanya Simuni, Fabrizio Stocchi, Alexander Storch, Indu Subramanian, Michele Tagliati, Michele Tinazzi, Jon Toledo, Yoshio Tsuboi, Richard Walker

**Affiliations:** 1 University of Cologne, Faculty of Medicine and University Hospital Cologne, Department of Neurology, Cologne, Germany; 2 Institute of Psychiatry, Psychology and Neuroscience, King’s College London, London, UK; 3 Department of Neurology, University Hospital Giessen and Marburg, Campus Marburg, Marburg, Germany; 4 Parkinson Foundation International Centre of Excellence, King’s College Hospital, London, UK; 5 University of Cologne, Faculty of Medicine and University Hospital Cologne, Department of Stereotactic and Functional Neurosurgery, Cologne, Germany; 6 Department of Neurology and Neurosurgery, Salford Royal NHS Foundation Trust, Manchester Academic Health Science Centre, Greater Manchester, UK; 7 Department of Neurosurgery, University Hospital Giessen and Marburg, Campus Marburg, Marburg, Germany; 8 Cognitive Neuroscience, Institute of Neuroscience and Medicine (INM-3), Research Centre Jülich, Jülich, Germany; 9 Parkinson and Movement Disorders Unit, Department of Neurosciences (DNS), University of Padua, Padova, Italy; 10 Center for Networked Biomedical Research in Neurodegenerative Diseases (CIBERNED), Carlos III Institute of Health, Madrid, Spain; 11 Departments of Psychiatry and Neurology, Perelman School of Medicine at the University of Pennsylvania, Philadelphia, PA 19104-2676 USA; 12 Department of Clinical and Movement Neurosciences, UCL Institute of Neurology, University College London, London, UK; 13 The Parkinson’s Disease and Movement Disorders Center, Department of Neurology, Mayo Clinic, Scottsdale, AZ USA; 14 Chulalongkorn Centre of Excellence for Parkinson’s Disease & Related Disorders, Department of Medicine, Faculty of Medicine, Chulalongkorn University and King Chulalongkorn Memorial Hospital, Thai Red Cross Society, Bangkok, Thailand; 15 Nuclear Medicine and PET, Aarhus University Hospital, Aarhus, Denmark; 16 Center for Neurodegenerative Diseases (CEMAND), Neuroscience Section, University of Salerno, Salerno, Italy; 17 Institute of Neuroscience, Newcastle University, Newcastle, UK; 18 Department of Nuclear Medicine and PET Centre, Aarhus University Hospital, Aarhus, Denmark; 19 King’s College London, Department of Psychology, London, UK; 20 Reviva Pharmaceuticals, Inc., Santa Clara, CA USA; 21 Faculty of Medicine and Dentistry, University of Plymouth, Plymouth, UK; 22 FAS Center for Systems Biology, Harvard University, Cambridge, MA USA; 23 Faculty of Medicine, Transilvania University of Brașov, Brașov, Romania; 24 Movement Disorder Clinic, University Hospital of Bispebjerg, Copenhagen, NV Denmark; 25 Oxford Parkinson’s Disease Centre, University of Oxford, Oxford, UK; 26 Nuffield Department of Clinical Neurosciences, University of Oxford, Oxford, UK; 27 Neurodegenerative Diseases Research Group, Institute of Pharmaceutical Sciences, Faculty of Life Sciences and Medicine, King’s College London, Newcomen Street, London, UK; 28 Department of Neurology, Seoul National University College of Medicine, Seoul, South Korea; 29 Division of Clinical Geriatrics, Department of Neurobiology, Care Sciences and Society, Center for Alzheimer Research, Karolinska Institutet, Stockholm, Sweden; 30 Department of Neurology, University Medical Centre Ljubljana, Ljubljana, Slovenia; 31 Parkinson’s Disease Service for the Older Person, Rankin Park Centre, John Hunter Hospital, HNELHD, Newcastle, NSW Australia; 32 Functional Movement Disorders Unit, Movement Disorders Program, Neurology Department, Hospital Ruber Internacional, Madrid, Spain; 33 Parkinson and Movement Disorders Unit, Department of Clinical Neurosciences, Fondazione IRCCS Istituto Neurologico Carlo Besta, Milan, Italy; 34 Parkinson’s Centre of Excellence at King’s College Hospital and King’s College London, London, UK; 35 Brain and Mind Centre, University of Sydney, Sydney, NSW Australia; 36 Department of Neurosciences Movement Disorders Center, University of California, San Diego, USA; 37 University of Kansas Medical Center, Kansas City, KS USA; 38 Department of Clinical Neurosciences, University of Calgary & Hotchkiss Brain Institute, Calgary, Canada; 39 Hurvitz Brain Sciences Program, Sunnybrook Research Institute, Toronto, ON Canada; 40 Department of Neurology, Osaka University Graduate School of Medicine, Osaka, Japan; 41 Parkinson Disease Research, Education, and Clinical Center, Philadelphia Veteran Affairs Medical Center, Philadelphia, PA USA; 42 Department of Neurology, University of Pennsylvania, Philadelphia, PA USA; 43 Department of Neurology, NYU School of Medicine, New York, NY USA; 44 University of Lund, Faculty of Medicine, Lund, Sweden; 45 Movement Disorders Unit, Sant Pau Hospital and Biomedical Research Institute (IIB-Sant Pau), Barcelona, Spain; 46 Neurology, National Hospital for Neurology & Neurosurgery, London, UK; 47 Newcastle Magnetic Resonance Centre & Positron Emission Tomography Centre, Newcastle University, Campus for Ageing & Vitality, Newcastle upon Tyne, UK; 48 Department of Neurology, Helsinki University Hospital, and Department of Neurological Sciences (Neurology), University of Helsinki, Helsinki, Finland; 49 Research Institute of McGill University Health Centre, Montréal, Canada; 50 Movement Disorders Clinic, National Institute of Neurology and Neurosurgery, Mexico City, Mexico; 51 Department of Neurology and Psychiatry, University of Santo Tomas Hospital, Manila, 1008 Philippines; 52 International Institute of Neuroscience, Saint Luke’s Medical Center, QUEZON, Philippines; 53 Center for Neurodiagnostic and Therapeutic Services, Metropolitan Medical Center, Manila, 1000 Philippines; 54 Department of Clinical Neurosciences, University College London (UCL) Institute of Neurology, Royal Free Campus, Rowland Hill Street, London, UK; 55 Department of Neurology, Northwestern University, Feinberg School of Medicine, Chicago, IL USA; 56 University and Institute for Research and Medical Care, IRCCS San Raffaele, Rome, Italy; 57 Division of Neurodegenerative Diseases, Department of Neurology, Dresden University of Technology, Dresden, Germany; 58 Department of Neurology, Dresden University of Technology, Dresden, Germany; 59 German Center for Neurodegenerative Diseases (DZNE), Research Site Dresden, Dresden, Germany; 60 UCLA/West LA VA, Los Angeles, CA USA; 61 Cedars-Sinai Medical Center, Los Angeles, CA USA; 62 Department of Neuroscience, Biomedicine, and Movement, University of Verona, Verona, Italy; 63 Department of Pathology & Laboratory Medicine, University of Pennsylvania, Philadelphia, PA USA; 64 Department of Neurology, Houston Methodist Hospital, Houston, TX USA; 65 Department of Neurology, Fukuoka University, Fukuoka, Japan; 66 Northumbria Healthcare NHS Foundation Trust, North Tyneside General Hospital, Rake Lane, North Shields, Tyne and Wear UK

**Keywords:** Parkinson's disease, Outcomes research

## Abstract

The effects of subthalamic nucleus deep brain stimulation (STN-DBS) on anxiety in Parkinson’s disease (PD) are understudied. We identified clinical predictors of STN-DBS effects on anxiety in this study. In this prospective, open-label, multicentre study, we assessed patients with anxiety undergoing STN-DBS for PD preoperatively and at 6-month follow-up postoperatively. We assessed the Hospital Anxiety and Depression Scale (HADS-anxiety and depression subscales), Unified PD Rating Scale-motor examination, Scales for Outcomes in PD-motor (SCOPA-M)-activities of daily living (ADL) and -motor complications, Non-Motor Symptom Scale (NMSS), PDQuestionnaire-8 (PDQ-8), and levodopa-equivalent daily dose. We tested changes at follow-up with Wilcoxon signed-rank test and corrected for multiple comparisons (Bonferroni method). We identified patients with a clinically relevant anxiety improvement of anxiety based on a designated threshold of ½ standard deviation of baseline HADS-anxiety. Moreover, we investigated predictors of HADS-anxiety changes with correlations and linear regressions. We included 50 patients with clinically relevant baseline anxiety (i.e., HADS-anxiety ≥ 8) aged 63.1 years ± 8.3 with 10.4 years ± 4.5 PD duration. HADS-anxiety improved significantly at 6-month follow-up as 80% of our cohort experienced clinically relevant anxiety improvement. In predictor analyses, worse baseline SCOPA-ADL and NMSS-urinary domain were associated with greater HADS-anxiety improvements. HADS-anxiety and PDQ-8 changes correlated moderately. Worse preoperative ADL and urinary symptoms predicted favourable postoperative anxiety outcome, which in turn was directly proportionate to greater QoL improvement. This study highlights the importance of detailed anxiety assessments alongside other non-motor and motor symptoms when advising and monitoring patients undergoing STN-DBS for PD.

## Introduction

Subthalamic nucleus (STN) deep brain stimulation (DBS) improves quality of life (QoL), and both motor and non-motor symptoms (NMS) in patients with Parkinson’s disease (PD)^1–3^. Present in 55.8% of patients with PD^4^, anxiety is one of the most prevalent NMS in PD. Literature shows that worse severity of anxiety is related to worse QoL and has an important impact on functioning in PD^5^. Unfortunately, anxiety is still underrecognised and undertreated in PD patients in clinical practice^6^. Previous studies have shown that neurodegeneration of the striatum and dopaminergic and serotonergic pathways is linked to anxiety in PD^7^. However, the exact pathomechanisms of anxiety in PD are still not fully understood. Nevertheless, there is Class I evidence for beneficial effects of STN-DBS on anxiety^8^. A meta-analysis has showed that the effects of STN-DBS on anxiety are highly heterogeneous across study cohorts^9^. Differences in baseline characteristics of study cohorts may contribute to the heterogeneity of results, and it is not clear if patient demographic and preoperative clinical parameters are predictors of postoperative anxiety outcomes.

We tested the hypotheses (1) that patients with PD undergoing STN-DBS with preoperative anxiety experience an improvement of anxiety and (2) that preoperative clinical predictors of the postoperative changes of anxiety can be identified. Furthermore, we explored the relationship between the postoperative changes of anxiety and QoL.

## Results

**Fig. 1:**
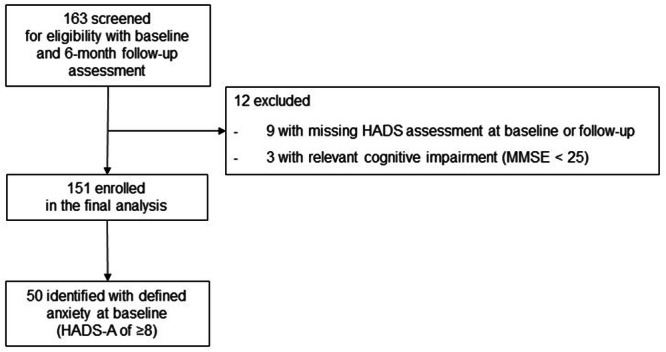
Flow chart of recruitment and data acquisition. DBS Deep Brain Stimulation, MMSE Mini Mental Status Examination, HADS Hospital Anxiety and Depression Scale.

In total, 163 consecutive PD patients were screened and underwent a 6-month follow-up postoperatively between August 2015 and March 2020. Of these, 151 patients (92 male) with a mean age of 61.5 years ± 8.7 and a mean disease duration of 10.4 years ± 4.7 were included in the final analysis (see Fig. 1). The cut-off for a clinically relevant change in HADS-A was 1.8 points (½ SD of HADS-A_baseline_ in the overall cohort, see Supplementary Table 1). In the overall cohort, 33.1% (50/151) of patients (27 male) scored ≥ 8 on the HADS-A at baseline and were classified as anxiety cohort. Their mean age was 63.1 years ± 8.3 and mean disease duration 10.4 years ± 4.5. Fewer patients scored ≥ 8 on the HADS-D at baseline (21.2% of patients, 32/151). None of the patients of our overall cohort fulfilled diagnostic criteria of anxiety disorders according to DSM-V or ICD-10 criteria during the course of our study.

### Clinical parameters at baseline and 6-month follow-up

**Fig. 2:**
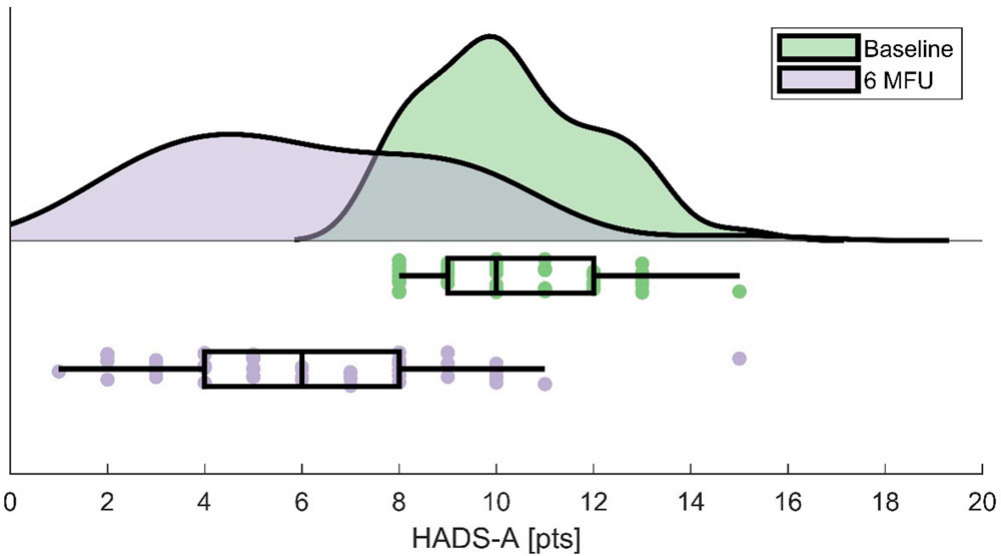
Hospital Anxiety and Depression Scale-anxiety subscale outcomes. Anxiety significantly improved at 6-month follow-up compared to baseline. The centre line illustrates median, the bounds of box represent the interquartile range (quartile 1 – quartile 3) and the whiskers extend to the furthest data point in each wing that is within 1.5 times the interquartile range. 6 MFU 6-month follow up, HADS-A Hospital Anxiety and Depression Scale-anxiety subscale, pts points.

Here we report the results of the anxiety cohort unless stated otherwise. HADS-A improved from baseline to follow-up (see Fig. 2). All other outcomes also improved at 6-month follow-up (see Table 1). The effect size was ‘large’ for HADS-A, HADS total, NMSS total, SCOPA-M motor complications, and LEDD total, ‘moderate’ for HADS-D, PDQ-8 SI, SCOPA-M ADL, and ‘small’ for LEDD-DA.

**Table 1:** Clinical characteristics at baseline and 6 months follow-up

	Baseline			6-month follow-up			Relative change [%]	Effect size	*p*-value
	*n*	mean	SD	*n*	mean	SD			
HADS total	50	17.6	3.9	50	11.8	5.5	33.0	1.22	**<** **0.001**
HADS-anxiety	50	10.3	1.7	50	6.1	3.0	40.8	1.72	**<** **0.001**
HADS-depression	50	7.3	2.8	50	5.7	3.5	21.9	0.50	**0.010**
PDQ-8 SI	50	41.9	17.9	50	31.9	15.0	23.9	0.61	**0.001**
NMSS total	50	84.4	40.4	50	53.8	30.7	36.3	0.85	**<** **0.001**
UPDRS-III	49	31.3	14.9	48	23.4	11.8	25.2	0.59	**0.001**
SCOPA-M ADL	49	8.4	3.1	50	6.4	3.6	23.8	0.60	**0.001**
SCOPA-M motor complications	49	6.1	2.7	49	3.6	2.4	41.0	0.98	**<** **0.001**
LEDD total	50	1085.0	534.7	49	595.5	323.5	45.1	1.11	**<** **0.001**
LEDD-DA	35	208.5	165.5	22	149.3	185.8	28.4	0.34	> 0.999

Wilcoxon signed rank or *t*-tests, when parametric test criteria were fulfilled, between baseline and 6-month follow-up to analyze within-group changes of clinical characteristics.

Bold font highlights significant results, *p* < 0.05.

All *p*-values are corrected for multiple comparisons using Bonferroni method.

We calculated relative change from baseline to follow-up [(mean Test_baseline_ – mean Test_follow-up_) / mean Test_baseline_ x 100] and quantified effect size with Cohen’s *d*.

Effect size: ‘small’ (0.20–0.49), ‘moderate’ (0.50–0.79), and ‘large’ ( ≥ 0.80).

*ADL* Activities of daily living, *HADS* Hospital Anxiety and Depression Scale, *LEDD* Levodopa equivalent daily dose, *LEDD-DA* LEDD of dopamine agonists, *NMSS* Non-Motor Symptom Scale, *PDQ-8 SI* Parkinson’s Disease Questionnaire-8 Summary Index, *SCOPA* Scales for Outcome in Parkinson’s Disease, *UPDRS-III* Unified Parkinson’s Disease Rating Scale-motor examination.

We observed a clinically relevant improvement in HADS-A in 80% of patients (40/50), a worsening of anxiety in 6% (3/50) and no clinically relevant anxiety change in 14% (7/50). Therefore, the NNT_anxiety improvement_ was 1.25. The proportion of patients who reported HADS-A ≥ 8 at 6-month follow-up was 18.5% (relative risk reduction: 44.1%, absolute risk reduction: 14.6%, NNT_anxiety remission_: 6.85).

Five patients were on a stable antidepressant medication regimen without changes of the drug or the dosage at baseline and 6-month follow-up. In three patients, an antidepressant medication was started between baseline and 6-month follow-up visits because of a depressive episode. None of the patients were treated with anxiolytic medication (e.g. benzodiazepines) between baseline and follow-up.

### Correlation analyses

Explorative correlation analyses between HADS-A change scores and clinical characteristics at baseline are shown in Table 2. These correlations were ‘moderate’ for baseline SCOPA-ADL and ‘weak’ for baseline HADS total and HADS-A. In partial correlations, we found these relationships to remain significant after controlling for baseline HADS-D. These partial correlations were ‘small’ for baseline SCOPA-ADL (*r* = 42, *p* = 0.003) and HADS-A (*r* = 0.30, *p* = 0.036). No significant correlations were found between HADS-A change score and age, disease duration, baseline NMSS domains and levodopa equivalent daily dose.

**Table 2:** Correlations between clinical characteristics at baseline and HADS-A change score anxiety cohort

	HADS-A change score		
	*n*	*r*	*p*-value
Age	50	−0.09	0.519
Disease duration	49	−0.19	0.198
HADS total	50	**0.31**	0.027
HADS-Anxiety	50	**0.33**	0.02
HADS-Depression	50	0.24	0.089
PDQ-8 Summary Index	50	0.12	0.413
NMSS total	50	0.14	0.344
UPDRS-III	49	0.24	0.105
SCOPA-M ADL	49	**0.45**	0.001
SCOPA-M motor complications	49	−0.05	0.742
LEDD total	50	−0.24	0.093
LEDD-DA	35	−0.16	0.355

Spearman correlations between HADS-A change score (baseline - 6-month follow-up) and clinical characteristics at baseline were calculated.

Bold font highlights significant results, *p* < 0.05; Positive correlations indicate that higher baseline values are associated with more postoperative improvement in anxiety.

*ADL* Activities of daily living, *HADS* Hospital Anxiety and Depression Scale, *LEDD* Levodopa equivalent daily dose, *LEDD-DA* LEDD of dopamine agonists, *NMSS* Non-Motor Symptom Scale, *PDQ-8 SI* Parkinson’s Disease Questionnaire-8 Summary Index, *SCOPA* Scales for Outcome in Parkinson’s Disease, *UPDRS-III* Unified Parkinson’s Disease Rating Scale-motor examination.

Furthermore, we explored the relationship between HADS-A change score and change scores of other clinical parameters, which was ‘moderate’ for the PDQ-8 SI (*r* = 0.47, *p* < 0.001) and ‘negligible’ or ‘weak’ for other parameters.

### Predictor analysis

Simple univariate linear regression analyses with HADS-A change score as the criterion variable were performed using candidate predictor variables identified in correlation analyses (relaxed threshold *p* < 0.2)^10,11^. This additionally included the following variables at baseline: HADS-D (*r* = 0.24, *p* = 0.101), disease duration (*r* = −0.19, *p* = 0.198), UPDRS-motor examination (*r* = 0.24, *p* = 0.105), NMSS urinary domain (*r* = 0.22, *p* = 0.117), and LEDD (*r* = −0.24, *p* = 0.093).

Simple univariate regression analyses with HADS-A change score at 6-month follow-up as the criterion variable were significant for the following independent variables: HADS total score (β = 0.36, *p* = 0.010), HADS-A (β = 0.40, *p* = 0.004), SCOPA-ADL (β = 0.46, *p* < 0.001), and NMSS domain urinary (β = 0.30, *p* = 0.033).

For the regression analyses, we excluded the variable HADS total score at baseline due to high intercorrelation with HADS-A at baseline (*r* = 0.70, *p* < 0.001). In the stepwise multiple regression analysis, the variables SCOPA-ADL and NMSS urinary domain remained significant. The multivariate multiple regression model accounted for 26.0% of the variance (*R*^2^_corr_ = 0.260) in HADS-A change score. In this model, SCOPA-ADL had the highest predictive value (β = 0.42, *p* = 0.002), followed by the NMSS urinary domain (β = 0.29, *p* = 0.028).

### Overall cohort

In the overall cohort, we observed a clinically relevant improvement of anxiety in the majority of patients with 47.0% (71/151) of patients and a worsening of anxiety in 16.6% (25/151) of patients, whereas the remaining 36.4% (55/151) showed no clinically relevant change. Clinical characteristics of the overall cohort at baseline and 6-month follow-up are presented in Supplementary Table 2. At 6-month follow-up, total scores of outcome parameters improved similar to the anxiety cohort. In contrast to the anxiety cohort, the HADS-D subscale did not improve in the overall cohort.

Explorative correlation analyses between HADS-A change score and clinical characteristics at baseline for the overall cohort are shown in Supplementary Table 3. In the overall cohort, baseline HADS-A predicted HADS-A changes at 6-month follow-up (β = 0.62, *p* < 0.001). The multivariate multiple model accounted for 37.7% of the variance in HADS-A change.

The reduction of LEDD total was similar in the overall cohort and in the anxiety cohort (46.0%, respectively 45.1%), whereas the reduction of LEDD dopamine agonists was 47.3% in the overall cohort and only 28.4% in the anxiety cohort.

## Discussion

In this prospective, open-label, multicentre study, we provide evidence that STN-DBS improves anxiety and that this improvement is associated with QoL improvement. We observed greater anxiety improvement in patients with worse baseline impairment of activities of daily living and urinary symptoms.

In line with the literature, we observed beneficial effects of STN-DBS on quality of life, motor and non-motor symptoms^1,12^.

In the overall cohort, 33.1% of patients reported to have anxiety at baseline according to the established HADS-A cut-off. This is consistent with previous DBS studies (40%) and below the reported prevalence in the general PD population (55.8% in a large study including 1072 patients)^4,13^. In line with a randomised, controlled study, we found a short-term improvement of anxiety measured with the HADS-A in our overall PD cohort^8^. Other studies have reported no change in anxiety using the State-Trait Anxiety Inventory (STAI) and the AMDP system (Association for Methodology and Documentation in Psychiatry) following bilateral STN-DBS, however both studies included small sample sizes of 27 and 15 patients respectively^14,15^. In our cohort of patients undergoing STN-DBS, we found that the number needed to treat for a clinically relevant anxiety improvement was lower than for other treatments of PD^16^.

As regards clinical predictors of postoperative anxiety outcome, the present study provides a pioneer report that worse baseline impairment of ADL and urinary dysfunction are linked to greater improvement of anxiety at 6-month follow-up postoperatively. Partial correlations showed that depression was not a confounding factor in the predictor analyses of postoperative anxiety changes.

Anxiety and urinary symptoms have a shared pathophysiology through serotonergic pathways^17,18^ and impairments of sensory gating^19,20^, which are improved by DBS^20,21^. An argument in favour of the shared pathophysiology is that in the overall cohort including patients with less severe anxiety, we observed no significant relationship between these parameters.

Furthermore, worse urinary symptoms and other autonomic dysfunction result in an increase of ADL impairments, which in turn results in worse anxiety^22^. In other words, ADL impairments mediate anxiety and urinary symptoms. In this context, in the general PD population, greater ADL impairments are related to worse anxiety^23^ and our current study extends this finding to a DBS cohort.

Furthermore, in the overall cohort of our study, we confirm findings of a previous study, which reported that worse baseline HADS-A is a predictor of greater postoperative HADS-A improvement^24^. This significant relationship was not reproduced in patients with baseline HADS-A ≥ 8, possibly due to a statistical effect resulting from a homogenisation of independent variable data in the analysis. Closely connected to this point, as opposed to the multivariate multiple regression model in the anxiety cohort, baseline HADS-A was included as a predictor variable in the overall cohort and contributed to increase the explained variance of HADS-A outcome from 26.0% in the anxiety cohort to 37.7% in the overall cohort.

The relationship of anxiety and dopaminergic medication needs further discussion: Non-motor fluctuations and OFF periods are associated with anxiety and this results in an improvement of anxious symptoms when dopaminergic medication is optimised^25^. In our cohort, we found that a reduction of LEDD total was not associated with the HADS-A change score. Therefore, the effect of STN-DBS goes beyond the amendment of total dopaminergic treatment. The reduction of LEDD of dopamine agonists was relatively small in the anxiety cohort. A possible explanation may be that dopamine agonists were tapered more cautiously in the anxiety cohort to prevent potential negative effects on mood symptoms, such as anxiety. Furthermore, our results show that LEDD total at baseline is not associated with change in anxiety.

Anxiety is related to sociodemographic parameters, motor symptoms, and quality of life. In line with the literature, we found a significant effect of STN-DBS on non-motor and motor symptoms, LEDD as well as QoL^1,2^. In line with previous studies we found that anxiety improvement was directly proportionate to QoL improvement^26^.

There are several limitations that need to be acknowledged. Firstly, our study included a relatively small sample size (*N* = 151 overall cohort and *N* = 50 anxious cohort). However, this is the only study investigating the effect of STN-DBS on anxiety in anxious patients at baseline. We addressed the effect of STN-DBS with a 6-month follow-up and, therefore, cannot conclude on the long-term effects of DBS on anxiety. Further longitudinal studies are required to explore this. Because clinically relevant neuropsychological and neuropsychiatric symptoms are considered contraindications for DBS treatment, baseline anxiety of patients in our study was mild to moderate and our observations need validation in a PD cohort with more severe anxiety. The HADS is useful for measuring the severity of anxiety^27^. However, assessments of further anxiety scales may provide information on the severity of specific aspects of anxiety.

Urinary symptoms were only assessed using the validated NMSS and a clinical rating scale for urinary symptoms and/or an objective detailed urinary assessment including an urodynamic study might have increased the accuracy of our findings. Nevertheless, the NMSS has been listed as “suggested” severity scale to assess urinary symptoms in PD^28^. Furthermore, we did not include a control group treated with best medical treatment and this should be considered in future studies. Closely connected to this point, a control group would help with the interpretation of the results of the number needed to treat analyses. Recently, we reported that more ventral locations of active DBS contacts are associated with greater improvement of anxiety^29^. In our present study, we have not looked into stimulation parameters as we were interested in clinical baseline characteristics, which might predict the anxiety outcome. In this context, we have not assessed anxiety in MedOFF/StimON state, which would help to discriminate between pure neurostimulation effects on anxiety in specific STN subregions, such as its limbic part, from possible effects of dopaminergic medication on anxiety. Another limitation of our study is the lack of genetic characterization of the patients. A previous study found no differences in anxiety prevalence and severity between GBA-associated PD and compared to idiopathic PD. However other mutations may influence anxiety prevalence and severity in patients and future studies should investigate the relationship of genetic mutations and non-motor and motor outcomes of DBS for PD.

In conclusion, we observed greater postoperative anxiety improvement in anxious patients with worse baseline impairment of ADL and urinary symptoms. Anxiety improvement was directly proportionate to QoL improvement.

This study emphasizes the importance of detailed preoperative motor and non-motor assessments and targeted measures to improve ADL and urinary symptoms in patients with anxiety undergoing STN-DBS for PD.

## Methods

### Study design and ethical approval

In this prospective, open-label, multicentre study we investigated patients undergoing STN-DBS with a 6-month follow-up^30–32^. Consecutive patients were screened between August 2015 and March 2020. Written informed consent was given by all patients prior to study inclusion. The study was conducted under the Declaration of Helsinki and was approved by local ethics committees (German Clinical Trials Register: DRKS00006735, Cologne study no.: 12-145; Marburg study no.: 155/17, UK: National Research Ethics Service Southeast London REC3-10/H0808/141, 000010084).

### Participants

PD diagnosis was established applying the UK Brain Bank criteria and patients were screened for DBS treatment according to the guidelines of the International PD and Movement Disorders Society^30,33,34^. DBS indication evaluations were conducted in a case-based approach including a multidisciplinary team of movement disorders neurologists, functional stereotactic neurosurgeons, psychiatrists experienced in DBS indication evaluations, speech and physiotherapists^35,36^. Patients with clinically relevant neuropsychological impairments or psychiatric diseases including anxiety disorders according to the current edition of the Diagnostic and Statistical Manual of Mental Disorders (DSM-5) or International Statistical Classification of Diseases and Related Health Problems (ICD-10) were not eligible for DBS treatment^37–40^. DBS surgical procedures are described elsewhere^41,42^.

### Clinical assessment

Clinical assessments were performed at preoperative baseline (MedON) and at 6-month follow-up after STN-DBS surgery (MedON/StimON)^43^. MedON was achieved at least 30 min after the first morning dose of levodopa when patients as well as movement disorder specialists noted clinical improvements^44^.

The main outcome was the anxiety subscale of the **Hospital Anxiety and Depression Scale (HADS)**:

The **HADS** is a 14-item self-report screening measure which is divided into a 7-item subscale for anxiety (HADS-A) and 7-item subscale for depression (HADS-D) and is commonly used in DBS studies^45,46^. Both subscales range from 0 (no anxiety/depression) to 21 (maximum anxiety/depression). For both HADS-A and HADS-D, scale developers proposed a cut-off value of ≥8 for possible cases^47^.

Furthermore, we assessed the following scales and parameters:

1. NMS: The **NMS Scale (NMSS)** is a clinician-rated tool containing 30 items divided into nine domains: 1) cardiovascular, 2) sleep/fatigue, 3) mood/apathy, 4) perceptual problems/hallucinations, 5) attention/memory, 6) gastrointestinal tract, 7) urinary, 8) sexual function, and 9) miscellaneous (including pain, inability to smell/taste, weight changes, and sweating) and records symptoms over the last four weeks. The NMSS is commonly used in DBS studies and its total score ranges from 0 (no NMS impairment) to 360 (maximum NMS impairment)^30,48,49^.
2. Motor disorder: Preoperative levodopa challenge tests were assessed with the **UPDRS-III**, which ranges from 0 (no impairment) to 108 (maximum impairment)^50^. Follow-up motor examination was assessed with the UPDRS-III (104 patients) or the Scales for Outcomes in PD-motor scale (SCOPA-M; 47 patients). The SCOPA-M was derived from the UPDRS, and the two scales highly correlate^51^. The SCOPA-M is commonly used for DBS studies and was chosen for time efficiency as its assessment time is approximately four times shorter than in the MDS-UPDRS^52,53^. Based on previously published conversion methods^51^, we report motor examination as UPDRS-III to simplify the interpretation of data. Activities of daily living (ADL) and motor complications were assessed with dedicated parts of the SCOPA-M. The SCOPA-M parts for motor examination, ADL, and motor complications range from 0 (no impairment) to 42, 21, and 12 (maximum impairment), respectively^51^.
3. QoL: **PD Questionnaire-8 (PDQ-8)** is a well-established self-reported tool to measure QoL in PD patients and is commonly used in patients undergoing DBS^11,54,55,56^. Furthermore, it is recommended by the International Parkinson and Movement Disorder Society^57^. The data are expressed as **PDQ-Summary Index (SI)** ranging from 0 (no QoL impairment) to 100 (maximum QoL impairment).
4. Finally, the **levodopa equivalent daily dose (LEDD)** was calculated according to the method by Jost et al. ^58^.

### Statistical analysis

Among the overall cohort undergoing STN-DBS we identified a subgroup of patients who experienced clinically relevant baseline anxious behaviour (cut-off value of HADS-A ≥ 8; hereinafter referred to as anxiety cohort).

Normality distribution of test scores was assessed using the Shapiro-Wilk method. Two-sided Wilcoxon signed-rank tests were employed to test for changes at 6-month follow-up. We corrected for multiple comparisons using the Bonferroni method and report adjusted *p*-values at the significance threshold of 0.05. We calculated relative change from baseline to follow-up [(mean Test_baseline_ – mean Test_follow-up_) / mean Test_baseline_ x 100] and quantified effect size with Cohen’s d^59^. Effect size: ‘small’ (0.20−0.49), ‘moderate’ (0.50−0.79), and ‘large’ ( ≥ 0.80).

Following a method reported previously^60^, we identified patients with a clinically relevant postoperative improvement of anxiety (‘anxiety responders’) based on a designated threshold of ½ standard deviation (SD) of HADS-A_baseline_ in the overall cohort. Patients who did not improve beyond this threshold were categorised as ‘anxiety non-responders’. In the anxiety cohort, we calculated the number needed to treat for a clinically relevant improvement of anxiety (NNT_anxiety improvement_ = 1/% of patients improving >1/2 SD of baseline HADS-A) and the number needed to treat for a remission of anxiety (NNT_anxiety remission_ = 1/absolute risk reduction of HADS-A ≥ 8 from baseline to 6-month follow-up).

Subsequently, we explored the relationship between HADS-A change scores (Test_baseline_ – Test_follow-up_) and preoperative demographic and clinical parameters using Spearman correlations. The correlations were categorised as following: 0.0−0.19 “very weak”, 0.20–0.39 “weak”, 0.40–0.59 “moderate”, 0.60–0.79 “strong” and 0.80−1.0 “very strong”.

To identify clinical predictors of anxious behaviour after 6 months of STN-DBS, simple univariate linear regressions with HADS-A change score as criterion variable were performed using candidate baseline predictor variables identified in correlation analyses (relaxed threshold *p* < 0.20)^10,11^. Partial correlations were used to control for confounding effects of depression assessed with the baseline HADS-D.

In a second step, significant predictors identified in the simple univariate regression analyses were included as candidate predictors in a stepwise multiple univariate regression analysis with HADS-A change score as criterion variable. Multi-collinearity was checked using intercorrelations between significant predictor variables in the simple linear regression (r < 0.6).

All analyses were conducted using Statistical Package for Social Science (SPSS version 28.0). The code for running the regression analyses is published at https://www.ibm.com/uk-en/analytics/spss-statistics-software.

### Reporting summary

Further information on research design is available in the Nature Research Reporting Summary linked to this article.

### Supplementary information

Supplementary file

Reporting Summary

## Data Availability

The data used to support the findings of this study are available from the corresponding author upon reasonable request (specification of a clear research question and preparedness to enter legal data-sharing agreements).
