# Supplementary material for: Predictors of short-term anxiety outcome in subthalamic stimulation for Parkinson’s disease
Source: NPJ Parkinsons Dis. 2024 Jun 8;10:114. doi: 10.1038/s41531-024-00701-6 (PMC11162430; doi:10.1038/s41531-024-00701-6)
Supplement: Supplementary file 1 — Supplementary file [file 41531_2024_701_MOESM1_ESM.docx]

**Supplementary Material**

**Supplementary Table 1 – Clinical characteristics in the overall cohort, anxiety-, and non-anxiety cohort at baseline**

|  |  | Overall cohort | |  | Anxiety cohort | |  | Non-anxiety cohort | |  |  |
| --- | --- | --- | --- | --- | --- | --- | --- | --- | --- | --- | --- |
|  |  | n | mean (SD) |  | n | mean (SD) |  | n | mean (SD) |  | *p* |
| HADS total |  | 151 | 10.9 (6.1) |  | 50 | 17.6 (3.9) |  | 101 | 7.5 (3.7) |  | **< 0.001** |
| HADS-anxiety |  | 151 | 6.0 (3.6) |  | 50 | 10.3 (1.7) |  | 101 | 3.9 (2.1) |  | **< 0.001** |
| HADS-depression |  | 151 | 4.8 (3.1) |  | 50 | 7.3 (2.8) |  | 101 | 3.6 (2.4) |  | **< 0.001** |
| PDQ-8 SI |  | 149 | 32.2 (16.3) |  | 50 | 41.9 (17.9) |  | 99 | 27.3 (13.0) |  | **< 0.001** |
| NMSS total |  | 150 | 61.3 (36.7) |  | 50 | 84.4 (40.4) |  | 100 | 49.7 (28.6) |  | **< 0.001** |
| UPDRS-motor examination |  | 149 | 26.7 (12.8) |  | 49 | 31.3 (14.9) |  | 100 | 24.5 (11.1) |  | **0.024** |
| SCOPA-M |  |  |  |  |  |  |  |  |  |  |  |
| Activities of daily living |  | 149 | 7.2 (3.2) |  | 49 | 8.4 (3.1) |  | 100 | 6.5 (3.0) |  | **0.005** |
| Motor complications |  | 149 | 5.2 (2.8) |  | 49 | 6.1 (2.7) |  | 100 | 4.8 (2.8) |  | 0.057 |
| LEDD total |  | 150 | 1120.4 (475.7) |  | 50 | 1085.0 (534.7) |  | 100 | 1138.1 (445.1) |  | > 0.999 |
| LEDD-DA |  | 113 | 255.9 (196.4) |  | 35 | 208.5 (165.5) |  | 78 | 277.1 (206.3) |  | 0.906 |

Mann-Whitney *U* tests or t-tests, when parametric test criteria were fulfilled, between the anxiety cohort and non-anxiety cohort regarding clinical characteristics at baseline.

Bold font highlights significant results, *p* < 0.05; All *p*-values are corrected for multiple comparisons using Bonferroni method.

**Abbreviations:**

HADS, Hospital Anxiety and Depression Scale; LEDD, Levodopa Equivalent Daily Dose; LEDD-DA, LEDD of Dopamine Agonists; NMSS, Non-Motor Symptom Scale; PDQ-8 SI, Parkinson’s Disease Questionnaire-8 Summary Index; SCOPA, Scales for Outcome in Parkinson’s Disease; UPDRS, Unified Parkinson’s Disease Rating Scale.

**Supplementary Table 2 Clinical characteristics at baseline and 6-month follow-up in the overall cohort**

|  | Baseline | | 6-month FU | | *p*-value |
| --- | --- | --- | --- | --- | --- |
|  | N | Mean (SD) | N | Mean (SD) |  |
| HADS total  HADS-Anxiety  HADS-Depression | 151  151  151 | 10.9 (6.1)  6.0 (3.6)  4.8 (3.1) | 151  151  151 | 9.1 (5.8)  4.6 (3.2)  4.5 (3.4) | **0.001**  **<0.001**  >0.999 |
| PDQ-8 SI | 149 | 32.2 (16.3) | 147 | 24.3 (15.7) | **<0.001** |
| NMSS total | 150 | 61.3 (36.7) | 151 | 42.0 (28.4) | **<0.001** |
| UPDRS-motor examination | 149 | 26.7 (12.8) | 142 | 19.5 (10.4) | **<0.001** |
| SCOPA-M  Activities  of daily living  Motor  complications  LEDD total  LEDD-DA | 149  149  150  113 | 7.2 (3.2)  5.2 (2.8)  1120.4 (475.7)  255.9 (196.4) | 150  149  149  87 | 5.4 (3.3)  3.0 (2.6)  605.0 (355.3)  135.0 (132.4) | **<0.001**  **<0.001**  **<0.001**  **<0.001** |
|  |  |  |  |  |  |

Wilcoxon signed rank tests between baseline and 6-month follow-up to analyze within-group changes of clinical characteristics.

Bold font highlights significant results, *p* < 0.05; All *p*-values are corrected for multiple comparisons using Bonferroni method.

**Abbreviations:** FU, Follow-up;

HADS, Hospital Anxiety and Depression Scale; LEDD, Levodopa Equivalent Daily Dose; LEDD-DA, LEDD of Dopamine Agonists; NMSS, Non-Motor Symptom Scale; PDQ-8 SI, Parkinson’s Disease Questionnaire-8 Summary Index; SCOPA-M, Scales for Outcome in Parkinson’s Disease-motor; UPDRS, Unified Parkinson’s Disease Rating Scale.

**Supplementary Table 3 Correlations between clinical characteristics at baseline and HADS-A change score in the overall cohort**

|  | HADS-A change score | | |
| --- | --- | --- | --- |
|  | N | *r* | *p*-value |
| HADS total  HADS-Anxiety  HADS-Depression | 151  151  151 | **0.59**  **0.66**  **0.38** | <0.001  <0.001  <0.001 |
| PDQ-8 Summary Index | 149 | **0.26** | 0.001 |
| NMSS total | 150 | **0.28** | <0.001 |
| Age  Disease duration | 151  150 | 0.04  -0.10 | 0.637  0.246 |
| UPDRS-motor examination | 149 | **0.17** | 0.039 |
| SCOPA-M  Activities of daily living  Motor complications | 149  149 | **0.17**  0.14 | 0.036  0.087 |
| LEDD total  LEDD-DA | 150  113 | -0.11  0.00 | 0.188  0.967 |
|  |  |  |  |

Spearman correlations between HADS-A change score (baseline - 6-month follow-up) and clinical characteristics at baseline were calculated.

Bold font highlights significant results, *p* < 0.05. Positive correlations indicate that higher baseline values are associated with more postoperative improvement in anxiety.

**Abbreviations:** HADS, Hospital Anxiety and Depression Scale; LEDD, Levodopa Equivalent Daily Dose; LEDD-DA, LEDD of Dopamine Agonists; NMSS, Non-Motor Symptom Scale; PDQ-8, Parkinson’s Disease Questionnaire-8; SCOPA-M, Scales for Outcome in Parkinson’s Disease-motor; UPDRS, Unified Parkinson’s Disease Rating Scale.

**Predictor analysis in the overall cohort**

Simple univariate linear regression analyses with change in HADS-A score as the criterion variable were performed using candidate predictor variables identified in correlation analyses (relaxed threshold p < 0.2).[3]

In the total cohort, this additionally included the following variables at baseline: SCOPA-motor complications (r = 0.14, p = 0.087), NMSS perceptual domain (r = 0.13, p = 0.109), and LEDD total (r = -0.11, p = 0.188), NMSS sexual dysfunction domain (r = 0.14, p = 0.100), and NMSS miscellaneous domain (r = 0.11, p = 0.177).

Simple univariate regression analyses with HADS-A change score at 6-month follow-up as the criterion variable were significant for the following independent variables: HADS total score (β = 0.57, p < 0.001), HADS-A (β = 0.63, p < 0.001), HADS-D (β = 0.39, p < 0.001), UPDRS-III (β = 0.21, p =0.012), SCOPA-ADL (β = 0.24, p = 0.003), and NMSS total (β = 0.35, p < 0.001), NMSS sleep domain (β = 0.32, p < 0.001), NMSS mood domain (β = 0.31, p < 0.001), NMSS memory domain (β = 0.27, , p < 0.001), NMSS gastrointestinal domain (β = 0.18, p = 0.032), NMSS sexual dysfunction domain (ß = 0.19, p = 0.023), and PDQ-8 SI (β = 0.32, , p < 0.001).

For the multiple univariate regression analysis, we excluded the variables HADS total score at baseline due to high intercorrelation with HADS-A at baseline (r = 0.91, p < 0.001) and NMSS total score due to high intercorrelation with NMSS sleep domain (r = 0.74, p < 0.001).

In the stepwise multiple regression analysis, the variable HADS-A baseline remained significant (ß = 0.62, p< 0.001). The multiple regression model accounted for 37.7% of the variance (*R*^2^_corr_ = 0.377) in HADS-A change score.

# Non-Motor Parkinson's Disease Study Group

Members of the MDS Non-Motor Parkinson's Disease Study Group listed here did not contribute to the current study.

Adler, Charles^13^Bhidayasiri, Roongroj^14^Borghammer, Per^15^
Barone, Paolo^16^
Brooks, David J.^17^
Brown, Richard^18^
Cantillon, Marc^19^
Carroll, Camille^20^
Coelho, Miguel^21^Falup-Pecurariu, Cristian^22^
Henriksen, Tove^23^
Hu, Michele^24^Jenner, Peter^25^Jeon, Beomseok^26^
Kramberger, Milica^27^
Kumar, Padma^28^
Kurtis, Mónica^29^Leta, Valentina^30^
Lewis, Simon^31^
Litvan, Irene^32^
Lyons, Kelly^33^
Martino, Davide^34^
Masellis, Mario^35^
Mochizuki, Hideki^36^
James F.Morley ^37^
Melissa Nirenberg^38^Odin, Per^39^
Pagonabarraga, Javier^40^
Panicker, Jalesh^41^
Pavese, Nicola^42^
Pekkonen, Eero^43^
Ron Postuma^44^Rodriguez Violante, Mayela^45^
Rosales, Raymond^46^
Anthony Schapira^47^Simuni, Tanya^48^
Stocchi, Fabrizio^49^Storch, Alexander^50^
Subramanian, Indu^51^
Tagliati, Michele^52^
Tinazzi, Michele^53^
Toledo, Jon^54^
Tsuboi, Yoshio^55^
Walker, Richard^56^

^13^ The Parkinson's Disease and Movement Disorders Center, Department of Neurology, Mayo Clinic, Scottsdale, Arizona, USA

^14^Chulalongkorn Centre of Excellence for Parkinson's Disease & Related Disorders, Department of Medicine, Faculty of Medicine, Chulalongkorn University and King Chulalongkorn Memorial Hospital, Thai Red Cross Society, Bangkok, Thailand

^15^ Nuclear Medicine and PET, Aarhus University Hospital, Aarhus, Denmark

^16^ Center for Neurodegenerative Diseases (CEMAND), Neuroscience Section, University of Salerno, Salerno, Italy

^17^ Institute of Neuroscience, Newcastle University, Newcastle, UK; Department of Nuclear Medicine and PET Centre, Aarhus University Hospital, Aarhus, Denmark

^18^ King's College London, Department of Psychology, London, UK

^19^ Reviva Pharmaceuticals, Inc., Santa Clara, CA, USA

^20^ Faculty of Medicine and Dentistry, University of Plymouth, Plymouth, UK

^21^ FAS Center for Systems Biology, Harvard University, Cambridge, MA, USA

^22^ Faculty of Medicine, Transilvania University of Brașov, Brașov, Romania

^23^ Movement Disorder Clinic, University Hospital of Bispebjerg, Copenhagen, NV, Denmark

^24^ Oxford Parkinson's Disease Centre, University of Oxford, UK; Nuffield Department of Clinical Neurosciences, University of Oxford, UK

^25^ Neurodegenerative Diseases Research Group, Institute of Pharmaceutical Sciences, Faculty of Life Sciences and Medicine, King's College London, Newcomen Street, London, UK

^26^ Department of Neurology, Seoul National University College of Medicine, Seoul, South Korea

^27^ Division of Clinical Geriatrics, Department of Neurobiology, Care Sciences and Society, Center for Alzheimer Research, Karolinska Institutet, Stockholm, Sweden Department of Neurology, University Medical Centre Ljubljana, Ljubljana, Slovenia

^28^ Parkinson's Disease Service for the Older Person, Rankin Park Centre, John Hunter Hospital, HNELHD, Newcastle, NSW, Australia

^29^ Functional Movement Disorders Unit, Movement Disorders Program, Neurology Department, Hospital Ruber Internacional, Madrid, Spain

^30^ Parkinson and Movement Disorders Unit, Department of Clinical Neurosciences, Fondazione IRCCS Istituto Neurologico Carlo Besta, Milan, Italy, and Parkinson's Centre of Excellence at King's College Hospital and King's College London, London, United Kingdom.

^31^ Brain and Mind Centre, University of Sydney, NSW, Australia

^32^ Department of Neurosciences Movement Disorders Center, University of California, San Diego, USA

^33^ University of Kansas Medical Center, Kansas City, KS, USA

^34^ Department of Clinical Neurosciences, University of Calgary & Hotchkiss Brain Institute, Calgary, Canada

^35^ Hurvitz Brain Sciences Program, Sunnybrook Research Institute, Toronto, ON, Canada

^36^ Department of Neurology, Osaka University Graduate School of Medicine, Osaka, Japan

^37^ Parkinson Disease Research, Education, and Clinical Center, Philadelphia Veteran Affairs Medical Center, Philadelphia, PA, USA; Department of Neurology, University of Pennsylvania, Philadelphia, PA, USA

^38^ Department of Neurology, NYU School of Medicine, New York, NY, USA

^39^ University of Lund, Faculty of Medicine, Lund, Sweden

^40^ Movement Disorders Unit, Sant Pau Hospital and Biomedical Research Institute (IIB-Sant Pau), Barcelona, Spain

^41^ Neurology, National Hospital for Neurology & Neurosurgery, London, United Kingdom

^42^ Newcastle Magnetic Resonance Centre & Positron Emission Tomography Centre, Newcastle University, Campus for Ageing & Vitality, Newcastle upon Tyne, United Kingdom

^43^ Department of Neurology, Helsinki University Hospital, and Department of Neurological Sciences (Neurology), University of Helsinki, Helsinki, Finland

^44^ Research Institute of McGill University Health Centre, Montréal, Canada

^45^ Movement Disorders Clinic, National Institute of Neurology and Neurosurgery, Mexico City, Mexico

^46^ Department of Neurology and Psychiatry, University of Santo Tomas Hospital, Manila 1008, Philippines; International Institute of Neuroscience, Saint Luke's Medical Center, Philippines; Center for Neurodiagnostic and Therapeutic Services, Metropolitan Medical Center, Manila 1000, Philippines

^47^ Department of Clinical Neurosciences, University College London (UCL) Institute of Neurology, Royal Free Campus, Rowland Hill Street, London, UK

^48^ Department of Neurology, Northwestern University, Feinberg School of Medicine, Chicago, IL , USA

^49^ University and Institute for Research and Medical Care, IRCCS San Raffaele, Rome, Italy

^50^ Division of Neurodegenerative Diseases, Department of Neurology, Dresden University of Technology, Dresden, Germany Department of Neurology, Dresden University of Technology, Dresden, Germany German Center for Neurodegenerative Diseases (DZNE), Research Site Dresden, Dresden, Germany

^51^ UCLA/West LA VA, Los Angeles, CA, United States

^52^ Cedars-Sinai Medical Center, Los Angeles, CA, United States

^53^ Department of Neuroscience, Biomedicine, and Movement, University of Verona, Verona, Italy

^54^ Department of Pathology & Laboratory Medicine, University of Pennsylvania, Philadelphia, PA, USA; Department of Neurology, Houston Methodist Hospital, Houston, TX, USA

^55^ Department of Neurology, Fukuoka University, Japan

^56^ Northumbria Healthcare NHS Foundation Trust, North Tyneside General Hospital, Rake Lane, North Shields, Tyne and Wear, United Kingdom
