# Supplementary material for: Predictors of short-term anxiety outcome in subthalamic stimulation for Parkinson’s disease
Source: NPJ Parkinsons Dis. 2024 Jun 8;10:114. doi: 10.1038/s41531-024-00701-6 (PMC11162430; doi:10.1038/s41531-024-00701-6)
Supplement: Supplementary file 2 — Reporting Summary [file 41531_2024_701_MOESM2_ESM.pdf]

Corresponding author(s): Dr. Haidar Dafsari

Last updated by author(s): Mar 15, 2024

Reporting Summary

Nature Portfolio wishes to improve the reproducibility of the work that we publish. This form provides structure for consistency and transparency in reporting. For further information on Nature Portfolio policies, see our Editorial Policies and the Editorial Policy Checklist.

Statistics

For all statistical analyses, confirm that the following items are present in the figure legend, table legend, main text, or Methods section.

n/a	Confirmed	
☐	☒	The exact sample size (*n*) for each experimental group/condition, given as a discrete number and unit of measurement
☐	☒	A statement on whether measurements were taken from distinct samples or whether the same sample was measured repeatedly
☐	☒	The statistical test(s) used AND whether they are one- or two-sided<br>*Only common tests should be described solely by name; describe more complex techniques in the Methods section.*
☐	☒	A description of all covariates tested
☐	☒	A description of any assumptions or corrections, such as tests of normality and adjustment for multiple comparisons
☐	☒	A full description of the statistical parameters including central tendency (e.g. means) or other basic estimates (e.g. regression coefficient) AND variation (e.g. standard deviation) or associated estimates of uncertainty (e.g. confidence intervals)
☐	☒	For null hypothesis testing, the test statistic (e.g. *F*, *t*, *r*) with confidence intervals, effect sizes, degrees of freedom and *P* value noted<br>*Give P values as exact values whenever suitable.*
☒	☐	For Bayesian analysis, information on the choice of priors and Markov chain Monte Carlo settings
☒	☐	For hierarchical and complex designs, identification of the appropriate level for tests and full reporting of outcomes
☐	☒	Estimates of effect sizes (e.g. Cohen's *d*, Pearson's *r*), indicating how they were calculated

Our web collection on statistics for biologists contains articles on many of the points above.

Software and code

Policy information about availability of computer code

Data collection	Data was collected in printed CRFs and then digitalized to SPSS version 28.0 for statistical analyses.
Data analysis	All analyses were conducted using Statistical Package for Social Science (SPSS version 28.0). The code for running the regression analyses is published at https://www.ibm.com/uk-en/analytics/spss-statistics-software.

For manuscripts utilizing custom algorithms or software that are central to the research but not yet described in published literature, software must be made available to editors and reviewers. We strongly encourage code deposition in a community repository (e.g. GitHub). See the Nature Portfolio guidelines for submitting code & software for further information.

Data

Policy information about availability of data

All manuscripts must include a data availability statement. This statement should provide the following information, where applicable:

- Accession codes, unique identifiers, or web links for publicly available datasets
- A description of any restrictions on data availability
- For clinical datasets or third party data, please ensure that the statement adheres to our policy

The data used to support the findings of this study are available from the corresponding author upon reasonable request.

Research involving human participants, their data, or biological material

Policy information about studies with human participants or human data. See also policy information about sex, gender (identity/presentation), and sexual orientation and race, ethnicity and racism.

Reporting on sex and gender	In total, 163 consecutive PD patients were screened and underwent a 6-month follow-up postoperatively between August 2015 and March 2020. Of these, 151 patients (92 male) with a mean age of 61.5 years ±8.7 and a mean disease duration of 10.4 years ±4.7 were included in the final analysis.
Reporting on race, ethnicity, or other socially relevant groupings	This study did not investigate race, ethnicity, or other socially relevant groupings.
Population characteristics	In total, 163 consecutive PD patients were screened and underwent a 6-month follow-up postoperatively between August 2015 and March 2020. Of these, 151 patients (92 male) with a mean age of 61.5 years ±8.7 and a mean disease duration of 10.4 years ±4.7 were included in the final analysis.
Recruitment	Consecutive patients undergoing bilateral subthalamic nucleus deep brain stimulation were screened between August 2015 and March 2020.
Ethics oversight	This study was approved by local ethics committees (German Clinical Trials Register: DRKS00006735, Cologne study no.: 12-145; Marburg study no.: 155/17, UK: National Research Ethics Service Southeast London REC3-10/H0808/141, 000010084).

Note that full information on the approval of the study protocol must also be provided in the manuscript.

Field-specific reporting

Please select the one below that is the best fit for your research. If you are not sure, read the appropriate sections before making your selection.

☒ Life sciences ☐ Behavioural & social sciences ☐ Ecological, evolutionary & environmental sciences

For a reference copy of the document with all sections, see nature.com/documents/nr-reporting-summary-flat.pdf

Life sciences study design

All studies must disclose on these points even when the disclosure is negative.

Sample size	In our overall cohort, 33.1% (50/151) of patients (27 male) scored ≥8 on the HADS-A at baseline and were classified as anxiety cohort. According to the "Rule of Ten", this sample size would have allowed a maximum of five preoperative predictor variables for postoperative anxiety outcome. In our results, we found four significant preoperative predictor variables. Therefore, the sample size was sufficient to detect all four predictor variables.
Data exclusions	Data from 12 out of 151 patients was excluded from the analysis (see Figure 1). Of these, 9 patients had missing HADS-anxiety (primary outcome parameter) assessments at baseline or follow-up and 3 patients had a clinically relevant cognitive impairment (Mini Mental State Examination <25 points).
Replication	Correlations, simple univariat linear rergessions, and stepwise linear regressions were employed to assess results based on different statistical tests. Additionally, the results of the anxiety cohort (n=50) were confirmed a larger sample (overall cohort, n=151).
Randomization	Randomization was not employed in our study. Age, disease duration, motor, and non-motor symptoms were covariates in our statistical analyses.
Blinding	Blinding was not relevant to our study as we investigated predictors of anxiety outcomes of deep brain stimulation and the primary outcome variable (HADS-anxiety) is a patient reported outcome.

Reporting for specific materials, systems and methods

We require information from authors about some types of materials, experimental systems and methods used in many studies. Here, indicate whether each material, system or method listed is relevant to your study. If you are not sure if a list item applies to your research, read the appropriate section before selecting a response.

Materials & experimental systems

n/a	Involved in the study
☒	☐ Antibodies
☒	☐ Eukaryotic cell lines
☒	☐ Palaeontology and archaeology
☒	☐ Animals and other organisms
☐	☒ Clinical data
☒	☐ Dual use research of concern
☒	☐ Plants

Methods

n/a	Involved in the study
☒	☐ ChIP-seq
☒	☐ Flow cytometry
☒	☐ MRI-based neuroimaging

Clinical data

Policy information about clinical studies

All manuscripts should comply with the ICMJE guidelines for publication of clinical research and a completed CONSORT checklist must be included with all submissions.

Clinical trial registration	DRKS00006735 (German Clinical Trials Register)
Study protocol	As a nonpharmacological and nondevice study according to German law and international standards (ICH-GCP) a study protocol is not required for this nonrandomized study.
Data collection	Consecutive patients were screened between August 2015 and March 2020.
Outcomes	The primary outcome variable was the Hospital Anxiety and Depression Scale (HADS) - anxiety subscale. This scale is recommended for use in clinical research by the Movement Disorders Society Clinical Outcomes Assessments Program Committee.

Plants

Seed stocks	not applicable (technical problem of the PDF form)
Novel plant genotypes	not applicable (technical problem of the PDF form)
Authentication	not applicable (technical problem of the PDF form)
